# Cognitive Competence as a Positive Youth Development Construct: A Conceptual Review

**DOI:** 10.1100/2012/210953

**Published:** 2012-04-24

**Authors:** Rachel C. F. Sun, Eadaoin K. P. Hui

**Affiliations:** Faculty of Education, The University of Hong Kong, Hong Kong

## Abstract

This paper focuses on discussing critical thinking and creative thinking as the core cognitive competence. It reviews and compares several theories of thinking, highlights the features of critical thinking and creative thinking, and delineates their interrelationships. It discusses cognitive competence as a positive youth development construct by linking its relationships with adolescent development and its contributions to adolescents' learning and wellbeing. Critical thinking and creative thinking are translated into self-regulated cognitive skills for adolescents to master and capitalize on, so as to facilitate knowledge construction, task completion, problem solving, and decision making. Ways of fostering these thinking skills, cognitive competence, and ultimately positive youth development are discussed.

## 1. Background

According to Piaget [1, 2], cognitive competence constitutes the cyclical processes of assimilation and accommodation, which indicates that people can manipulate their personal experiences as well as organize and adapt their thoughts to guide their behavior. Similarly, Fry [3] pointed out that cognitive competence comprises three interwoven and interdependent components: cognitive structures, cognitive processes, and overt behaviors. Among them, “cognitive processes,” such as metacognition, cognitive styles of self-regulation, and cognitive skills of thinking, reasoning, analyzing problems, and information processing, can affect one's “behaviors” like task performance, problem solving, and decision making, as well as “cognitive structures,” such as self-schemas and goal orientation. It further points out that people can make a difference in their cognitive development and capability by manipulating their mental processes and cognitive styles via using appropriate thinking skills. It is also argued that cognitive competence is more than an ability to manipulate and strategize information, but an ability to internalize, self-regulate, and transfer these cognitive skills to construct knowledge and make sense of the surroundings [4, 5].

In the literature, there are various types of thinking, for instance, logical thinking and reasoning [1, 2], legislative, executive, and judicial thinking styles [6], synthetic, analytic and practical intellectual skills [7], divergent thinking and evaluative thinking [8–10], and lateral thinking, and vertical thinking [11]. There are also important features of adolescent thinking, for instance, being able to think abstractly, test hypotheses, conduct reasoning, and make causal inferences [1, 2]. All these are used to facilitate knowledge construction, task completion, problem solving, and decision making, but their application commonly requires critical thinking and creative thinking. Indeed, numerous studies have demonstrated that adolescents who were equipped with critical thinking and creative thinking had better academic performance [12, 13], health [14, 15], cognitive development [16], psychosocial development [17], and identity development [18] and were less likely to engage in unhealthy or problem behavior [19, 20]. Therefore, both critical thinking and creative thinking are regarded as generic transferable life skills for adolescents [11, 21–23], who have to deal with various developmental stresses and challenges, such as puberty changes, adjustments in social roles and expectations, school transition, examination, pursuit of further studies, preparing for or entering the labor market, expansion of social circles, and development of romantic relationship. Nonetheless, there are also situations that adolescents still engage in problem behaviors even though they understand the pros and cons or make numerous imaginative solutions of which none of them are realistic to solve the problems. Therefore, it is of paramount importance to guide adolescents to master the thinking skills well in order to foster learning [24, 25], leadership [26–28], and positive youth development [29, 30].

In regard of this, the present paper focuses on discussing critical thinking and creative thinking as the core cognitive competence. It reviews and compares several theories of thinking, highlights the features of critical thinking and creative thinking, and delineates their interrelationships. It discusses cognitive competence as a positive youth development construct by linking its relationships with adolescent development and its contributions to adolescents' learning, wellbeing and positive development. It shows how critical thinking and creative thinking can be translated into self-regulated cognitive skills for adolescents to master and capitalize on to achieve better task performance, generate precise solutions to problems, and make right decisions. It is believed that these thinking skills not only facilitate life-long learning and holistic development among youngsters, but also prepare youngsters to be the future masters of the society who are able to solve social problems and contribute to global development.

## 2. Definition of Cognitive Competence

There are broad definitions of cognitive competence [1–5], as well as narrow definitions [29]. Building on the definition given by Sun and Hui [29], the present paper refers critical thinking and creative thinking as the core cognitive competence, though it is noted that cognitive competence includes, but is not limited to these two thinking. Critical thinking refers to reasoning and making inferences, and creative thinking means stretching one's spectacles, evaluating multiple ideas and alternatives, and generating novel and practical ideas. The definitions of critical thinking and creative thinking, and the specific cognitive skills involved are reviewed in the followings.

### 2.1. Critical Thinking

According to Paul [31], “critical thinking is the intellectually disciplined process of actively and skillfully conceptualizing, applying, analyzing, synthesizing, and/or evaluating information gathered from, or generated by, observation, experience, reflection, or communication, as a guide to belief and action” (page 22). Moreover, “critical thinking refers to the use of cognitive skills or strategies that increase the probability of a desirable outcome. Critical thinking is purposeful, reasoned, and goal-directed. It is the kind of thinking involved in solving problems, formulating inferences, calculating likelihoods, and making decisions” (page 70) [32]. Therefore, critical thinking is a process that activates certain cognitive skills so as to make the best judgments regarding on what to believe and what to do [33].

“Reason” and “inference” are the two main cognitive skills in critical thinking [34], that are used when making judgments or decisions, accepting beliefs, and developing ideas and alternatives. It is important to make good and objective reasons for one's beliefs, by recognizing one's subjective point of view, gathering multiple and diverse points of view, coordinating various views (including those for and against the concerned issues), for generating sufficient reasons and reliable evidence before making a judgment [34, 35]. Since there are no explicit guidelines for judging what sufficient and reliable reasons are, it may run the risk of developing under- or overcritical judgments. Therefore, rational thinking is needed [35]. Lipman [36] further elaborated that when engaging in critical thinking, one should make reference to reliable, strong, and relevant criteria, such as norms, shared values, laws, rules, definitions, facts, and values, and pay attention to the situational factors, such as special circumstances and limitations, and variations in culture, context, time, and people. One should also be reflective and self-correcting so as to question one's own thoughts, identify the errors in one's own thinking, and then make reasonable corrections. In other words, critical thinking means one needs to be critical to the concerned issues as well as one's thinking, so that one can proceed to make inference and deduction from the information collected for doing a rational evaluation and making a reasonable decision [34]. Paul [31] added that critical thinkers like to reason about their reasoning and make inferences and conceptualization with rational justification. Their habitual inspection of the thinking is, in fact, “an action of ongoing creation” contributing to their cognitive and intellectual advancement. In sum, critical thinking includes the skills of reasoning and making inferences, and it is both evaluative and productive [37] that encompasses the ideas of rationality and creativity, respectively [38, 39].

### 2.2. Creative Thinking

Creative thinking refers to thinking that is novel and that produces ideas that are of value [40]. According to Sternberg [6, 7], creative thinking is autonomous and people can choose to capitalize on certain “thinking styles” and “intellectual skills” to maximize their creativity [41, 42]. Among the thirteen thinking styles, research findings showed that five of them, including legislative, judicial, hierarchical, global, and liberal (i.e., type I intellectual styles) are related to creative thinking [43, 44]. Adolescents choose to regulate their thinking processes and behaviors accordingly can thus learn to master creative thinking. Therefore, it is preferable that, adolescents, when performing a task, can evaluate the task (*judicial *thinking style) and choose to develop their own ideas, rules, and procedures (*legislative *thinking style), instead of simply following rules and instructions (executive thinking style). When doing multiple tasks, adolescents can rank things in priority and distribute attention to the tasks in accordance with the value of the tasks (*hierarchical* thinking style). Besides drilling the details of a task (local thinking style), adolescents can also look at the overall picture of the task (*global *thinking style). Moreover, adolescents can be proactive in choosing works involving novelty and ambiguity (*liberal *thinking style). All these are in parallel with the synthetic, analytic, and practical intellectual skills for solving problems [7], in which creative people would interpret problems in a new way and avoid being bounded by conventional thinking (synthetic skills), identify the most valuable and novel idea (analytic skills), and make out ways to demonstrate the values of that idea (practical skills). In short, creative thinking refers to the cognitive skills of stretching one's spectacles, generating and evaluating multiple ideas and alternatives, and generating novel and practical ideas. Similarly, creative thinking (the components of judicial thinking style and analytic skills) entails critical thinking, because adolescents have to be skeptical enough to criticize their own ideas so as to initiate positive changes in their thinking. It is believed that after continuously practicing these thinking styles and skills, adolescents would learn to welcome changes and innovations, to think globally and progressively rather than conservatively, and become habitual in generating novel and realistic ideas that help task completion, problem solving, and decision making.

## 3. Relationship between Creative Thinking and Critical Thinking

Conceptually, creative thinking and critical thinking are not dichotomous and conflicting [7, 31, 45]. Both of them operate together productively to leading to creative and effective problem solving, just as “divergent thinking” and “evaluative thinking” do [8–10, 44, 46]. Adolescents are activating creative thinking when they use divergent thinking to generate numerous and diverse solutions to a problem, in which they redefine problems in novel ways that other people usually do not see (originality), select relevant information to conceptualize a problem (flexibility), draw an analogy between the old problem and the new interpretation, and combine the information in a novel way (fluency) [47, 48]. To find out the most sensible novel solution, adolescents also activate evaluative and critical thinking to perform valuation. Likewise, creative thinking and critical thinking are comparable to de Bono's conceptions of “lateral thinking” and “vertical thinking” [11, 49], in which the former requires people to see things from multiple perspectives and arrive at the solutions from new angles, whereas the later requires people to see things sequentially and conventionally and generate solutions from a deeper investigation. He highlighted that both thinking are equally important in generating novel and practical ideas for problem solving, because solutions generated by lateral thinking solely are not realistic enough for tackling problems, whereas solutions generated by vertical thinking lack novelty for energizing progressive advancement though the problem is practically solved. Some empirical studies also revealed that both creative thinking and critical thinking (or divergent thinking and evaluative thinking, or lateral thinking and vertical thinking) are complementary with each other in effective problem solving and decision making [50, 51].

 Research findings also showed that both critical thinking and creative thinking are closely related to each other to facilitate learning and knowledge construction [52]. In learning, simply recalling the facts and information are usually being accused of a straight-forward surface approach. However, it is argued that recalling is a step to build up a solid foundation of knowledge, so that one can further execute the higher-order cognitive processes of critical thinking and creative thinking to understand the meanings of the information and to apply the learnt knowledge to daily life situations [53]. To further constructing one's own knowledge and meaningful learning, more sophisticated critical thinking skills are indispensable for analyzing (such as differentiating, organizing, and attributing) and evaluating (e.g., checking and critiquing) multiple information, followed by using creative thinking to create (such as generating, planning, and producing) knowledge with originality and novelty. Paul [31] stressed that “the creative dimension of thinking is best fostered by joining with the critical dimension” (page 21).

It demonstrates that there is a close linkage between critical thinking and creative thinking in problem solving and learning, and therefore acquiring and mastering of these thinking skills are of paramount importance. Adolescents should be encouraged to utilize these thinking skills effectively, not simply to get problems solved and to know more, but to achieve effective problem solving and meaningful knowledge construction.

## 4. Antecedents of Cognitive Competence

There are various factors, such as heredity, environmental stimuli, socioeconomic status, culture, and maturation, contributing to adolescents' cognitive competence [54]. Among them, the role of cognitive development and maturation is indispensable. According to Piaget [1, 2], one's cognitive competence becomes sophisticated throughout four developmental stages according to one's age. Children aged between 7 and 11 years are at the concrete operational stage. Their logical reasoning is developed which allows them to mentally arrange and compare things. Critical thinking starts to blossom as their thinking becomes decentered and less egocentric, which allows them to consider others' perspectives and clarify one's thoughts [1, 55]. This logical and critical thinking becomes advanced when they reach the formal operational stage (age 12 or above) because they are able to think systematically, manipulate mental objects, test hypotheses, and draw conclusions based on reasoning. It reveals that developmental age and maturation are related to the development of cognitive competence, and at the same time, adolescents' cognitive competence is changing progressively via their active manipulation of the mental processes.

 Meaningful social interaction is another factor helping adolescents excel cognitively. Vygotsky [4, 5] believed that through conversation, collaboration, modeling, guidance and encouragement, adolescents learn better ways of thinking, reasoning and solving problems from more competent peers and adults, when compared with performing the task alone. Creative imagination and thinking also become more sophisticated during adolescence, when youngsters actively use private speech to conceptualize their own ways of problem solving from those learnt from social models [56]. Empirical findings also showed that students were cognitively advanced when they could internalize, self-regulate, and transfer these cognitive skills, so as to complete the tasks independently without the help of the others [52].

Sociocultural contexts and settings, for example, family, classroom, school, and educational system, also account for cognitive competence among adolescents. Thus, another critical antecedent of cognitive competence is whether there is “mediated learning experience” that provides the opportunities for adolescents (i) to learn the thinking skills, and (ii) to become aware of these thinking skills and processes that help them to excel in task performance, and also become more self-regulatory and self-efficacious in transferring the skills to wider contexts. There are many research findings demonstrated that structured programs, activities, scaffolding instructions and guidance, and social interactions are effective in helping children and adolescents to equip and transfer these thinking skills. For instance, the Philosophy for Children Program in training critical thinking [21], the Purdue Creative Thinking Program in training divergent thinking [20, 23], and the de Bono Cognitive Research Trust Program for Creative Thinking (CoRT Program) in training lateral thinking and vertical thinking [11] which could facilitate the fluency, flexibility, and originality of thinking  [50, 51]. Mushrooming evidences also showed the potential of incorporating creative thinking in classroom teaching for mainstream students [24, 25] and outside classroom context among gifted students [26, 27] for them to transfer the skills to independent learning and problem solving.

## 5. Cognitive Competence and Adolescent Developmental Outcomes

With reference to the holistic development of adolescents, there are interconnections and reciprocal influences among cognitive, moral, behavioral, emotional, social, physical, aesthetical, and spiritual domains. Hence, cognitive competence is vital in contributing to adolescent development in specific domains as well as their holistic wellbeing. In education, critical thinking was revealed to play a crucial role in students' self-regulatory learning by influencing their mastery of learning goals and deep information processing [57]. Some studies also found that critical thinking significantly predicted students' academic performance [12, 13]. Apart from the positive effects on intellectual development, health education research studies showed that strengthening adolescents' critical thinking skills was one of the important components that enabled students' autonomy in identifying their health needs and making healthy choices [14], developing healthy body image and preventing disordered eating patterns [19]. Critical thinking was also found to help adolescents to be more pragmatic about media messages and thus less likely to internalize some distorted messages regarding beauty standard [15] and had lower intention of substance use in the future [20].

In addition, compared with those having lower levels of creative thinking, adolescents having higher levels of creative thinking were found to have higher levels of internal control and self-acceptance [58], lower levels of depression and more likely to adopt a positive attributional style [59]. A series of research studies, which were mainly conducted with Chinese university students by Zhang and her colleagues also demonstrated that creativity-generating styles (i.e., type I intellectual style) were positively related to academic achievement [60–62], self-esteem [63] and emotion management [64], and contributory to cognitive development [16], psychosocial development [17, 65], and identity development [18]. The long-term positive effects of creative thinking was also demonstrated, as an 18-year longitudinal research study found that creative thinking and creative performance, rather than school grade at adolescence were better predictors of life accomplishment in adulthood [66].

All these show that critical thinking and creative thinking are the developmental assets and strengths. Adolescents who are equipped with these thinking skills tend to have better learning, wellbeing, and positive development. In regard of these beneficial effects on adolescent development, promotion of cognitive competence in education (e.g., [67, 68]) and developmental programs aiming at preventing youth problems and promoting healthy growth (e.g., [29, 69]) have been advocated over recent decades. Taking Hong Kong as an example, nurturing students' independent and critical thinking and creativity is clearly spelt out in the objectives of the senior secondary education and higher education [70], for such thinking skills are believed to be indispensable generic skills helping students to learn how to learn, and so as to become independent life-long learners. In addition, cognitive competence is regarded as one of the core psychosocial competencies facilitating adolescent holistic development in a curricula-based positive youth development program adopted by numerous secondary schools in Hong Kong since 2005 [30].

## 6. Fostering Cognitive Competence in Adolescents

To foster cognitive competence among adolescents, one of the ways is to introduce creative thinking and critical thinking skills and provide social opportunities for adolescents to master these skills. The central issues are to let students to understand “What are these practical skills?”, “How can they be carried out?”, and “Why do I use these skills?” so as to help them to internalize, self-regulate, and transfer the learnt skills. It can be done explicitly or implicitly, both inside and outside schools, in the following three ways.

### 6.1. Direct Teaching (Bolt-On Approach)

Thinking skills can be taught explicitly to students in context-free situation. For instance, the instrumental enrichment aims at developing students' generic thinking skills that enable their ability to solve problems and transfer their problem solving skills to a wider context [71]. As aforementioned, there are many programs targeting at training students' critical and creative thinking skills, for example, Philosophy for Children Program [21], the Purdue Creative Thinking Program [22, 23], and the CoRT Program [11]. In addition, thinking skills can also be directly introduced in developmental programs, like leadership training [26, 27] and positive youth development program [29, 30, 72], in which students' cognitive competence are fostered and sharpened leading to the forward flow of positive developmental attributes, and vice versa. In such kind of direct teaching, teachers play a crucial role in a series of structural “mediated learning experiences” to guide students to master the skills in defining problems, developing plans and strategies, and transferring the classroom learning to other life aspects. As there is a spiral of learning to think and thinking to learn, arranging more opportunities for students to practice, reflect and evaluate the skills is necessary for them to assimilate, accommodate, internalize, and advance and transfer the thinking strategies and processes.

### 6.2. Embedded Approach

Embedded approach means that thinking skills are taught and practiced within a subject in school formal curriculum, for example, in Social Studies [73], liberal studies [70], and Sciences [24, 25]. This approach allows students to apply critical and creative thinking skills in a meaningful subject context, and at the same time, to develop a deep understanding of the subject matters through utilizing the skills. “Inquiry teaching” [74, 75] can be adopted, in which students are enabled to evaluate existing information and proceed to construct new knowledge of that subject. In the learning process, reasoning skills are emphasized and students are guided to form hypotheses, test hypotheses, make predictions, select cases, distinguish consider alternative hypotheses, examine misconceptions in their current reasoning, ask questions, and challenge authorities. Moreover, probing questions and dialoging can stimulate and challenge students' thoughts, sharpen their skills and motivation to reason, to make inferences, and even to generate creative and valuable ideas.

At the same time, “problem-based learning” can be incorporated. The problems need to be novel, ambiguous, or challenging, so as to generate cognitive conflicts and stimulate higher-order thinking [1]. In other words, the problems need to be structured with reference to the students' prior knowledge in that subject areas and existing levels of thinking skills, with the purpose to progress students' generic skills of critical thinking and creative thinking in analyzing and solving the problems. Collins and Stevens [74] noted that, “by turning learning into problem solving, by carefully selecting cases that optimize the abilities the teacher is trying to teach, by making students grapple with counterexamples and entrapments, teachers challenge the students more than by any other teaching method. The students come out of the experience are able to attack novel problems by applying these strategies themselves” (page 229). Therefore, the students can become more skillful, esteemed, and motivated to master the thinking skills inside and outside their school learning.

### 6.3. Infusion Approach

Infusion means having the subject matters and thinking skills learnt together across curriculum. There is no specific lessons design to teach thinking skills, but teachers plan and deliver lessons with an emphasis on thinking, and to let students developing the feelings of competence and autonomy via self-regulation that encourages them to transfer the mastered skills across different subject areas and life situations. The overarching goal is to let student master these generic and transferable skills, take the responsibility in self-regulatory learning, and become a person with independent thinking. An example is the project of Activating Children's Thinking Skills [52] for primary school children in Northern Ireland, in which metacognitive skills of critical thinking, creative thinking, searching for meaning, problem solving, and decision making are infused across curriculum, demonstrated with significant effects on students' cognitive advancement as well as social and behavioral improvement. However, the infusion approach cannot succeed without structured pedagogy, for instance, engaging students in open-ended activities, collaborative activities, classroom dialogue, and joint meaning making [76] are some strategies of social construction of learning [4, 5]. To help students to transfer thinking skills to other tasks, teachers can also give examples or ask students to generate examples, so as to guide them of how these forms of reasoning, inference-making and idea-generating can be applied inside the subject areas as well as outside. Paul and his colleagues [77, 78] have given detailed suggestions of how critical thinking and creative thinking can be incorporated into teaching and curriculum.

## 7. Conclusion

In this paper, cognitive competence is defined as critical thinking and creative thinking skills which facilitate effective problem solving, decision making, and learning for positive youth development. However, there are several conceptual and research gaps that need to be filled. First, as the narrow definition was adopted, further review is needed to elucidate the broad conception of cognitive competence. Second, although the literature showed that both critical thinking and creative thinking are interrelated thinking skills, more empirical research on their relationships is needed. Third, there were studies showing that critical thinking and creative thinking are beneficial to adolescents' cognitive advancement, psychosocial wellbeing, life-long learning, and accomplishment. However, most of these were separate studies. Further research is needed to demonstrate their unique effects as well as their interactive effects on adolescents' problem solving, decision making, learning, and development. Lastly, while three ways are discussed to promote adolescents' cognitive competence, it is necessary to have more vigorous research studies to evaluate and compare the effectiveness of these approaches across age groups and cultural settings. It is hoped that tailor-made curriculum or programs can be offered to cater to the unique characteristics and needs of adolescents for their cognitive advancement and positive development.
